# Dietary Supplementation of Vitamin E and α-Lipoic Acid Upregulates Cell Growth and Signaling Genes in Rat Myocardium

**Published:** 2006-12

**Authors:** Susan A. Marsh, Steven Mason, Leigh C. Ward, Jeff S. Coombes

**Affiliations:** 1 *School of Human Movement Studies, The University of Queensland, Brisbane 4072, Australia;*; 2 *School of Molecular and Microbial Sciences, The University of Queensland, Brisbane 4072, Australia*

**Keywords:** antioxidants, vitamin E, α-lipoic acidgene expression, myocardium

## Abstract

The efficacy of antioxidant supplementation in the prevention of cardiovascular disease appears equivocal, however the use of more potent antioxidant combinations than those traditionally used may exert a more positive effect. We have shown previously that supplementation of vitamin E and α-lipoic acid increases cardiac performance during post-ischemia reperfusion in older rats and increases Bcl-2 levels in endothelial cells. The purpose of this study was to examine the effects of vitamin E and α-lipoic acid supplementation on myocardial gene expression with a view to determine their mechanism of action. Young male rats received either a control (n=7) or vitamin E and α-lipoic acid supplemented diet (n=8) for 14 weeks. RNA from myocardial tissue was then amplified and samples were pooled within groups and competitively hybridized to 8.5K oligonucleotide rat microarrays. The relative expression of each gene was then compared to the control sample. Animals that received the antioxidant-supplemented diet exhibited upregulation (>1.5×) of 13 genes in the myocardium with 2 genes downregulated. Upregulated genes include those involved in cell growth and maintenance (*LynB, Csf1r, Akt2, Tp53*), cell signaling (LynB, Csf1r) and signal transduction (*Pacsin2, Csf1r*). Downregulated genes encode thyroid (*Thrsp*) and F-actin binding proteins (*Nexilin*).

## INTRODUCTION

The efficacy of antioxidant supplementation for the primary prevention of cardiovascular disease appears equivocal. This may be due, in part, to the wide range of antioxidants studied, doses used, and the populations from which study participants are drawn and the use of synergistic combinations of antioxidant compounds. We have previously demonstrated that supplementation with moderate doses of vitamin E and α-lipoic acid increases cardiac performance during post-ischemia reperfusion in older rats (1) and upregulates Bcl-2 protein levels in left ventricular (LV) endothelial cells (2). α-lipoic acid is a potent water-soluble antioxidant that is rapidly taken up by the cell and reduced to dihydrolipoic acid (DHLA) which is active as both an intracellular and extracellular antioxidant (3). The small amount of DHLA remaining in the cell enhances the recycling of other antioxidants such as vitamin E, glutathione and ascorbate (3, 4). Vitamin E is a major chain-breaking, lipid soluble antioxidant that resides in cellular membranes thus, the combination of α-lipoic acid and vitamin E provides antioxidant protection in both the aqueous and lipid phases of the cell. In addition, both compounds have potent non-antioxidant properties including blood clotting (5-7), glucose transport system (8) and various cell signaling pathways (9-11) that may be unrelated to their antioxidant roles. The purpose of this study was to examine the effect of vitamin E and α-lipoic acid supplementation on myocardial gene expression in the left ventricle.

## METHODS

### Animals

This experiment was approved by The University of Queensland Animal Ethics Committee in accordance with National Health and Medical Research Council guidelines. Male Wistar rats, aged four weeks obtained from the Central Animal Breeding House, The University of Queensland, were randomly assigned to receive either a standard laboratory rodent diet (n=7) or an antioxidant-supplemented diet (n=8) for 14 weeks. Animals that received the antioxidant diet were fed the same rat chow as the non-supplemented groups but with 1000 IU vitamin E (d-α-tocopherol succinate, Covitol 1185, Cognis, Melbourne, Australia) and 1.6 g α-lipoic acid (Lipoec, Cognis, Melbourne, Australia) added per kg of diet. The dietary antioxidants and dose rates used in this study were based on previous results from our laboratory (1, 2, 5). Rats were housed 2-3 per cage, maintained on a 12-12 h light/dark cycle and provided with rat chow and tap water *ad libitum*.

### Microarray

Total RNA was extracted from the left ventricles using the TRIzol^®^ method (Invitrogen, Melbourne, Australia). RNA samples were further purified using an RNeasy mini kit (Qiagen, Doncaster, Australia). The mRNA was reverse transcribed to cDNA with direct incorporation of cyanine dyes. The RNA from each tissue was compared to the expression profile of a “common control” consisting of a pool of RNA from several individual animals. Following labelling, each case sample was competitively hydridised with the common control to 5K MWG oligonucleotide rat microarrays (Ramaciotti Centre, University of NSW, Australia). Microarrays were scanned and the data analysed using Gene Spring GX software (Agilent Technologies, Palo Alto, CA).

### Antioxidants

Concentrations of vitamin E (α-tocopherol) were determined in left ventricular tissue and plasma by reverse-phase high performance liquid chromatography (HPLC) using the liquid-liquid extraction method of Taibi and Nicotra (12). Briefly, proteins were precipitated and lipids extracted in a single step by incubation with an ethanol-chloroform mixture (3:1 v/v). After separation of the precipitated protein, 50 μl supernatant were injected onto a LiChrospher C18 column (250 × 4 mm, 5 μm; Merck, Darmstadt, Germany) with a flow rate of 1 ml.min^-1^ and 9 MPa backpressure and analysed using fluorometric detection. Stock solutions of dl-α-tocopherol (Fluka, Buchs, Switzerland) were used as external standards. In our hands, the coefficient of variation for this assay is <2%.

Tissue and plasma concentrations of α-lipoic acid were not measured as previous data in our laboratory indicated that the compound was undetectable in both control and supplemented animals (2, 5) as α-lipoic acid is rapidly converted to various metabolites also with antioxidant properties (13, 14). This finding is consistent with previous studies (15).

### Statistical analysis

The relative expression of each gene compared to common control was determined followed by the variance of each genes expression relative to the common control within each group and finally the statistical difference in relative expression between the groups. Initial filters selected only genes which show statistically different expression between the groups with either over-expression of greater than 1.5 or less than 0.7 fold. Plasma parameters were analysed using an independent t-test and values reported are means ± SEM. Significance was established at *P*<0.05.

## RESULTS

Fourteen weeks of antioxidant supplementation resulted in an increase in plasma levels of vitamin E compared to non-supplemented animals (25.1 ± 2.3 vs. 36.0 ± 2.6 μM respectively; *P*<0.05). No such change was evident in myocardial tissue (3.70 ± 0.32 vs. 3.65 ± 0.41 mol/g protein; *P*>0.05)

Antioxidant supplementation upregulated (>1.5×) genes in the myocardium associated with cell growth and signalling (Table 1). Upregulated genes that negatively control cell growth include *Tp53* (tumour suppressor (p53) gene), *Madh5* (Smad5), *Klf15* (Kruppel-like transcription factor) and *Lyn* (Lyn B tyrosine kinase) whereas *Akt2* (protein kinase B) and *Csf1r* (CSF-1 receptor) genes are associated with cell growth and proliferation. In addition, *Pacsin2*, a known regulator of protein kinase C, was increased. Antioxidant supplemented rats also exhibited a significant increase in *Renbp* (renin-binding protein) and *Lypla1* (calcium-independent phospholipase A2). Downregulated genes encode thyroid (*Thrsp*) and F-actin binding proteins (*Nexilin*).

**Table 1:** Myocardial gene expression in rats following 14 weeks of antioxidant supplementation^a^

Gene	Product	Expression vs. control (control=1)

Csf1r	CSF-1 receptor	4.9
Lyn	Lyn B tyrosine kinase	3.9
Lypla1	lysophospholipase 1	3.8
Renbp	renin-binding protein	3.0
Klf15	Kruppel-like factor 15	1.9
Pacsin2	Protein kinase C and casein kinase substrate in neurons 2 protein	1.7
Tp53	p53	1.6
Madh5	Smad5	1.6
Akt2	Akt2	1.6
Nexilin	F-actin binding protein b-Nexilin	0.58
Thrsp	thyroid hormone responsive SPOT14 homolog	0.36

a Only those genes whose expression was either greater than 1.5 or less than 0.7 fold that of the common control are listed.

## DISCUSSION

Reactive oxygen species (ROS) are known to play an integral role in a number of cell signalling pathways and an imbalance between ROS production and antioxidant protection (oxidative stress) can cause and/or contribute to various disease processes. In addition, several antioxidants can function in non-antioxidant capacities which can also affect cell protection and signalling pathways. In this study, we have shown that vitamin E and α-lipoic acid supplementation activates various cell signaling genes associated with the Akt pathway as *Akt2, Tp53, Lyn, CSF1r* and *Madh5/Smad5* all of which were upregulated in the myocardium of supplemented animals. We recently reported an increase in Bcl-2 protein in endothelial cells of rats following the same supplementation regime (2), suggesting a protective role of the combination of vitamin E and α-lipoic acid. The Akt pathway is known to be pro-survival (16) and the increase in *Akt2* supports our previous findings. *Akt* activation phosphorylates Murine double minute 2 (Mdm2), a potent inhibitor of the pro-apoptotic protein, p53 (17). p53 levels can be increased directly by *Tp53* upregulation and indirectly by *Lyn* via increased translocation of p53 to the cytoplasm and reversal of Mdm2-mediated degradation of p53 (18). Similarly, the increase in *Madh5* is likely related to the increase in *Tp53* as the gene product of *Madh5*, Smad5, plays an integral role in the TGF-β signaling pathway and inhibits apoptosis via a p53-mediated pathway (19). The upregulation of *CSF1r* in the myocardium also increases intracellular Lyn and Akt activity (20). We have previously shown increases in lipid peroxidation *in vivo* with vitamin E and α-lipoic acid supplementation (2) and an increase in caspase-3 activity with increasing concentrations of α-lipoic acid with no concomitant rise in DNA fragmentation (11). We therefore speculate that the upregulation of the cell signaling genes in the current study is likely the result of an increase in oxidative stress-mediated activation of p53 production although as both vitamin E and α-lipoic acid have known non-antioxidant properties, it is also possible that these changes are mediated via other pathways.

The physiological significance of a modest increase in *Renbp* is unclear as the product, renin-binding protein, is a cytoplasmic protein and is unlikely to function in binding circulating renin. However, renin-binding protein has been shown to control the availability of betaN-acetyl-glucosamine (GlcNAc) (21). Glycosylation of proteins by O-linked GlcNAc is a cell signaling and transduction pathway enhanced during hyperglycemia and diabetes that appears to function in a similar manner to phosphorylation. α-lipoic acid supplementation is known to provide a beneficial effect in diabetic patients via mechanisms including attenuation of hyperglycemia (22), suppression of advanced glycation end production formation (23) and improved insulin sensitivity (24). Thus, the increase in *Renbp* expression with vitamin E and α-lipoic acid supplementation may have a number of functional ramifications in cell signaling pathways, particularly with diabetes.

In this study we have shown a significant upregulation of cell signaling genes associated with both pro- and anti-apoptotic pathways following supplementation with vitamin E and α-lipoic acid. These antioxidants have been demonstrated to act in a synergistic manner when given together and we have previously reported contradictory findings with an increase in the anti-apoptotic protein Bcl-2 *in vivo* and an increase in caspase-3 activity *in vitro*. Myocardial tissue is composed of four major cell types, cardiomyocytes, fibroblasts, endothelial cells and vascular smooth muscle cells, that all play a major role in LV function. Data from the current study complements the findings of previous work by Haramaki and colleagues (25, 26) who demonstrated an increase in myocardial protection with vitamin E and α-lipoic acid supplementation and we are currently investigating the effects of supplementation on individual cell types in the LV. The limited amounts of tissue available for further analysis prevented us from using RT-PCR; future experiments in our laboratory will seek to confirm these findings and to determine if these genes play a role in myocardial cell signaling and protection.
